# Coronary Artery Calcification on Non-Cardiac Gated CT Thorax Scans: A Single Tertiary Centre Retrospective Observational Study

**DOI:** 10.3390/jcdd12120480

**Published:** 2025-12-04

**Authors:** Robert S. Doyle, Divyanshu Jain, Patrick Devitt, Jack Hartnett, Hugo C. Temperley, Catherine McGorrian

**Affiliations:** 1 Department of Cardiology, Mater Misericordiae University Hospital, Eccles Street, Dublin 7, D07 WXC8 Dublin, Ireland; 2 Department of Radiology, St James Hospital, James’s St, Saint James, Dublin 8, D08 NHY1 Dublin, Ireland

**Keywords:** coronary artery calcification, incidental findings, non-gated CT thorax, cardiovascular risk management, retrospective cohort study, inpatient care

## Abstract

Background: While the 2024 ESC Guidelines provide guidance on utilising incidental CAC findings from non-gated CT scans to enhance risk stratification and guide treatment decisions, there remain gaps in detailed protocols for managing such incidental findings, particularly in inpatient settings. An incidental finding of CAC in a patient without known atherosclerosis provides an opportunity to assess cardiac risk, promote risk factor optimisation and evaluate need for further cardiac work up. The aim of this study was to assess the prevalence of incidental coronary artery calcification on non-cardiac dedicated gated CT thorax scans among general medical inpatients and to evaluate the subsequent management of these findings. Methods: This was a single-centre retrospective observational study of consecutive general medical inpatients aged 40–75, who had undergone a non-cardiac gated CT thorax during their admission, between February and March 2025. Data were collected using local electronic health records. Exclusion criteria were patients with known ischaemic heart disease (IHD). Risk factor assessment was noted by documentation of smoking status, hypertension, diabetes and low-density lipoprotein (LDL) values. Results: A total of 186 patients with thoracic CT scans were identified. On review of all CT reports, 53 (28.4%) patients had CAC reported, of whom 17 had known IHD. Therefore 36 (19.4%) patients were identified for further analysis. An exercise stress test was booked in none of the patients. A coronary angiogram was booked in 1 patient. Conclusions: One fifth of medical inpatients in our study had a new finding of CAC on thoracic imaging. Cardiovascular risk factors of LDL and HbA1c were checked in less than half of patients. None of these patients went on to have functional testing. There is a valuable opportunity to optimise cardiac risk factors and evaluate the need for functional testing in a subset of patients with CAC reported on non-cardiac CTs. This can be facilitated by raising awareness and implementing a flowchart tool for hospital physicians to reference.

## 1. Introduction

Cardiovascular disease (CVD) remains a leading cause of morbidity and mortality worldwide, with coronary artery disease (CAD) accounting for a significant proportion of disease burden [1,2]. Coronary artery calcification (CAC) is a well-established marker of subclinical atherosclerosis, strongly associated with future cardiovascular events [3,4]. Dedicated cardiac imaging modalities, such as CT coronary angiography (CTCA) and electrocardiogram (ECG)-gated CT calcium scoring, provide quantitative assessment of CAC and guide risk stratification and management according to established guidelines from the European Society of Cardiology (ESC) and the American Heart Association/American College of Cardiology (AHA/ACC) [5,6]. These guidelines recommend lifestyle modifications, pharmacotherapy (statins, aspirin), and further investigations (functional testing or invasive angiography) based on CAC severity and patient risk profile [5,7]. Furthermore, in patients with established chronic coronary syndromes, an updated meta-analysis of randomised controlled trials has demonstrated that percutaneous coronary revascularization in addition to optimal medical therapy significantly reduces cardiovascular mortality and angina severity compared with medical therapy alone [8].

However, CAC is frequently detected incidentally on non-cardiac gated CT thorax scans performed for unrelated indications, such as suspected pulmonary embolism (PE), malignancy, aortic dissection, or sepsis [9,10]. Non-gated CT thorax scans, which lack ECG synchronisation, do not allow for formal Agatston scoring but can still identify CAC qualitatively or semi-quantitatively [11,12]. Despite its prognostic value, incidental CAC on these scans is often underreported or overlooked in clinical practice, leading to missed opportunities for preventive interventions [13,14]. Studies have shown that incidental CAC on non-gated chest CT is prevalent in 20–65% of patients and correlates with increased CVD risk, independent of traditional risk factors [15,16,17]. Yet, while the 2024 ESC Guidelines provide some guidance on using incidental CAC findings from previous non-gated chest CT scans to enhance risk stratification and inform treatment decisions, such as integrating with risk models to determine the need for functional testing, there remain gaps in standardised protocols for reporting and managing such findings, particularly in inpatient settings where acute care priorities may overshadow preventive opportunities [2,18,19].

This retrospective observational study aims to explore the prevalence of incidental CAC reported on non-cardiac gated CT thorax scans in medical inpatients without known ischaemic heart disease (IHD) and to evaluate screening and optimisation of cardiovascular risk factors in these patients.

## 2. Methods

### 2.1. Study Design and Population

**Figure 1:**
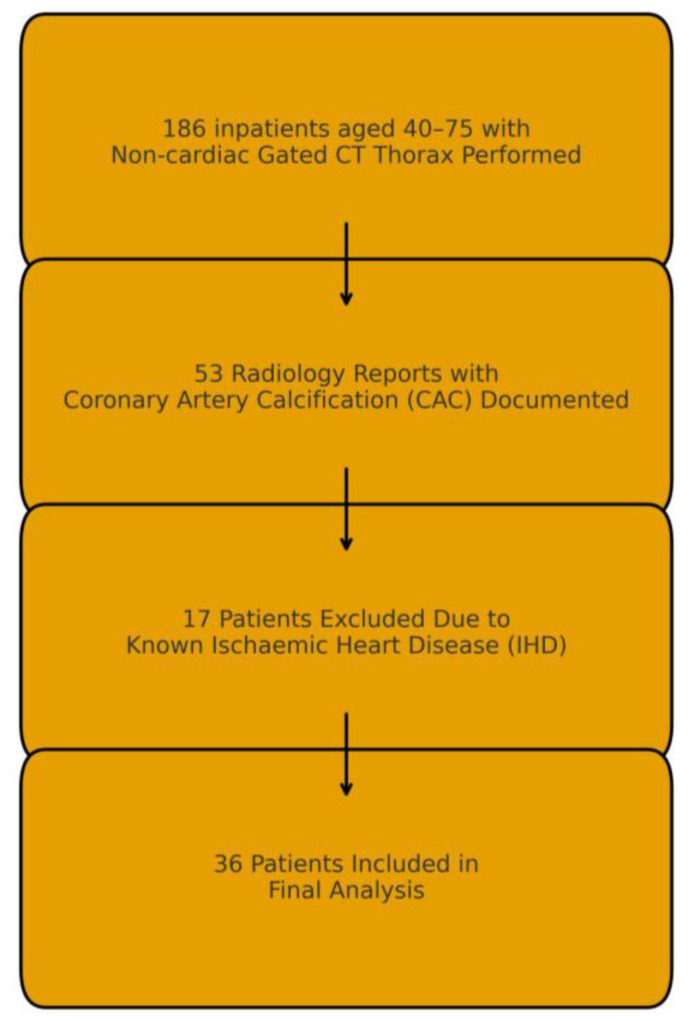
Patient flow diagram showing identification and inclusion of patients with incidental coronary artery calcification on non-gated CT thorax.

This was a single-centre observational cohort study conducted at the Mater Misericordiae University Hospital, a tertiary referral centre in Dublin, Ireland. The study adhered to the Strengthening the Reporting of Observational Studies in Epidemiology (STROBE) guidelines [20], as outlined in the Supplementary Materials. We reviewed consecutive unscheduled general medical admissions via the Emergency Department between 1 February 2025, and 31 March 2025, who were aged 40–75 years and underwent a non-cardiac gated CT thorax during their admission (Figure 1). This age range was selected based on guidelines recommending opportunistic CAC assessment in middle-aged adults at intermediate or higher CVD risk [5,6]. Data were collected retrospectively over a 4-week period from May to June 2025.

Inclusion criteria: non-contrast or contrast-enhanced CT thorax performed for non-cardiac indications with a completed consultant radiologist report. Exclusion criteria: known IHD (prior myocardial infarction, PCI, CABG or documented CAD on previous imaging) and age outside 40–75 years.

### 2.2. Data Collection

Data were extracted from the available patient documents, including patient letters and discharge summaries, CT orders and reports, and blood results and other testing requested, using a standardised protocol to ensure consistency. Collected variables included the following: Demographics, including age and sex, were recorded for all participants. The primary clinical indication for undergoing CT thoracic imaging was documented. Coronary artery calcium (CAC) reporting was assessed based on its presence as noted in the radiology report, using a qualitative description categorised as present or absent. Cardiovascular risk factors were evaluated through documentation of blood pressure (with hypertension defined as office systolic blood pressure [SBP] > 140 mmHg or diastolic blood pressure [DBP] > 90 mmHg on two occasions [5]), LDL cholesterol (considered suboptimal if >1.8 mmol/L [5]), smoking status (classified as active smoker), and HbA1c (deemed suboptimal if >48 mmol/mol or >6.5% for diabetes diagnosis [21]). For each risk factor, we determined whether it was recorded, suboptimally controlled, and treated if abnormal through antihypertensive initiation, statin prescription, or smoking cessation advice. Downstream investigations following CAC detection were tracked, including the booking of exercise stress tests, CT coronary angiography (CTCA), or invasive coronary angiography.

CT scans were interpreted by consultant radiologists using standard protocols for non-gated thorax imaging, with CAC noted incidentally if visible in the coronary arteries [11]. CAC was reported qualitatively as present or absent; no ordinal severity grading (mild/moderate/severe) or Agatston scoring was provided in any report. Although ordinal visual scoring is recommended by SCCT/STR guidelines even on non-gated scans to aid risk stratification [11], reliable Agatston calcium scoring is not possible on standard non-cardiac gated CT thorax scans. A dedicated ECG-gated acquisition protocol is required to minimise cardiac motion artefact and enable accurate, accurate quantification [11]. No dedicated workstation for Agatston scoring was used, aligning with real-world practice for non-cardiac scans.

### 2.3. Data Analysis

Descriptive statistics were used to summarise data. Continuous variables (e.g., age) were reported as means. Categorical variables (e.g., sex, risk factor prevalence) are expressed as frequencies and percentages. No inferential statistics were performed due to the exploratory nature of the study. Missing data for cardiovascular risk factors were addressed by conducting analyses solely on available records without imputation, as the study focused on descriptive summaries of documented assessments.

### 2.4. Ethical Considerations

The study was approved by the Mater Misericordiae University Hospital Research Audit Committee. Patient data were anonymised, and informed consent was waived due to the retrospective design and minimal risk.

## 3. Results

A total of 186 patients meeting the age criteria underwent non-cardiac gated CT thorax scans during the study period. CAC was reported in 53 (28.4%) patients, of whom 17 had known IHD and were excluded. Thus, 36 (19.4%) patients with incidental CAC and no prior IHD were included for analysis.

Baseline demographics and clinical indications are summarised in Table 1. The mean age was 63 years (SD = 8), with a male predominance (72%). The most common indication for thoracic CT was CTPA for suspected PE (55%), followed by CT thorax for malignancy (28%).

**Figure 2:**
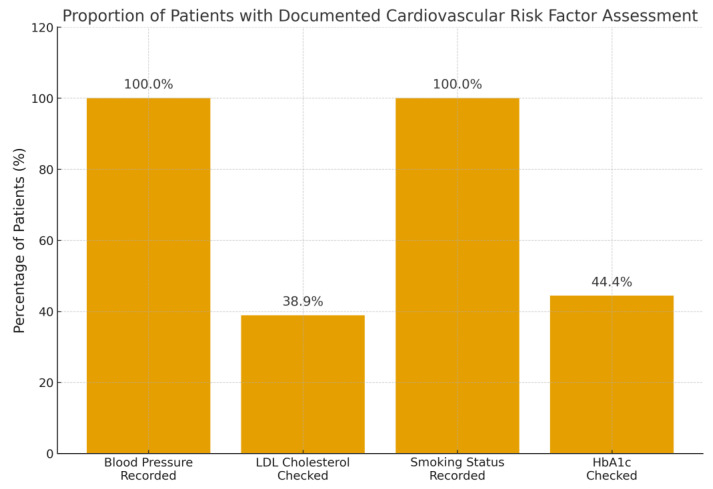
Proportion of the 36 patients with incidental coronary artery calcification in whom key cardiovascular risk factors were documented during the index admission.

Cardiovascular risk factor assessment is detailed in Table 2. Blood pressure and smoking status were universally recorded (100%), but LDL and HbA1c were checked in only 39% and 44% of patients, respectively. The proportion of patients in whom each key cardiovascular risk factor was documented is illustrated in Figure 2. Among those with recorded values, suboptimal control was common (e.g., 64% for blood pressure), with low rates of treatment initiation (e.g., 26% for hypertension, 50% for dyslipidemia). For smoking status (recorded in 100%), “suboptimal control” was defined as active/current smoking (38.9% of the cohort) and “treated if suboptimal” indicates that smoking cessation advice, pharmacotherapy, or referral to cessation services was documented (35% of active smokers).

No patients were booked for a functional cardiac investigation, such as an exercise stress test. One patient (2.8%) was scheduled for coronary angiography based on clinical judgement beyond CAC alone. No patients underwent dedicated ECG-gated coronary artery calcium scoring (Agatston score) or CT coronary angiography as a consequence of the incidental CAC finding.

## 4. Discussion

This retrospective study demonstrates a notable prevalence of incidental CAC in almost a fifth (19.4%) of medical inpatients without known CAD identified on non-cardiac gated CT thorax scans. This rate aligns with the NOTIFY-1 project by Sandhu et al., which identified CAC in 20.1% of 2113 patients undergoing non-gated chest CT [14]. Other reports in the literature reporting rates of 20–50% in similar cohorts [15,16,22]. Furthermore, the overall detection rate in our study of 28.4% of reports identifying CAC before study exclusions is consistent with systematic reviews and meta-analyses estimating incidental CAC in 35–45% of non-gated chest CTs performed for non-cardiac indications [17,23].

The demographic profile of our cohort, predominantly males (72%), with a mean age 63 years, mirrors populations at higher atherosclerotic risk, as evidenced by studies showing increased CAC prevalence with age and male sex [3,24,25]. Indications such as suspected PE (55%) and malignancy (28%) reflect common inpatient scenarios where incidental CAC may be overlooked amidst acute management priorities [26]. The prognostic implications of incidental CAC are well-documented; it independently predicts cardiovascular events, all-cause mortality, and stroke risk, even in asymptomatic individuals [13,27,28,29]. CAC screening provides prognostic value for all-cause mortality beyond traditional risk factors in hospital inpatients [13]. Similarly, Hariharan et al. (2024) reported predating incidental CAC in 84.8% (39/46) of ACS patients undergoing angiography, highlighting missed preventive opportunities [30]. A meta-analysis by Peng et al. (2023) found CAC on non-gated CT associated with hazard ratios of 1.51 (95% CI: 1.28–1.79) for all-cause death, 1.57 (95% CI: 1.33–1.84) for death/MI/stroke, and 1.69 (95% CI: 1.45–1.98) for death/MI/stroke/revascularization compared with CAC = 0 in adults without known ASCVD; patients with CAC ≥ 100 had a 10-year ASCVD risk of 24%, with only 26% on statins [23]. Incidental CAC ≥ 100 Agatston units correlates with elevated mortality beyond traditional risk scores like QRISK3 or Framingham [4,31,32].

Despite this, our results reveal significant gaps in risk factor management. Blood pressure and smoking status were routinely documented. However, LDL and HbA1c assessments were infrequent, 39% and 44%, respectively, with suboptimal control in most cases and low treatment rates, with only 50% of patients with dyslipidaemia receiving statins. This under-management echoes other retrospective studies; for example, Balakrishnan et al. (2017) reported coronary calcification identified in 69% of reports when present, with limited follow-up on preventive therapy [18]. CAC is frequently not reported on non-gated thoracic CT [33,34,35,36], and a recent survey in 2022 showed that only 17% of non-cardiothoracic radiologists in Canada were aware of the correlation between CAC scores on gated and non-gated thoracic CT [36]. A quality improvement project by Sandhu et al. (2022) demonstrated that notifying clinicians of incidental CAC increased statin prescriptions [14]. Similarly, Huey et al. (2024) showed that educating radiologists and modifying reporting templates improved incidental CAC reporting and quantification [37]. The absence of functional testing in our cohort is concerning, as ESC guidelines recommend further evaluation in intermediate-risk patients with CAC [2]. Only one patient proceeded to angiography, possibly due to competing acute illnesses or lack of awareness.

Computed tomography has become a cornerstone of modern cardiology practice, extending far beyond opportunistic risk stratification to sophisticated procedural planning. In complex coronary interventions, pre-procedural coronary CT angiography enables detailed characterisation of calcium morphology using a novel seven-point scale ranging from “spot” (≤10% cross-sectional involvement) to “full moon” (circumferential, 100% cross-sectional involvement) [38]. Similarly, in structural heart interventions such as transcatheter aortic valve replacement, CT-guided commissural alignment significantly reduces the risk of coronary ostia overlap and preserves future coronary access [39].

No patients in our cohort underwent dedicated ECG-gated calcium scoring or CT coronary angiography as a direct result of the incidental CAC finding. This is most likely due to the acute inpatient setting, qualitative-only reporting, absence of angina symptoms, and lack of standardised pathways. This represents a potentially missed opportunity not only for risk factor optimisation but also for identification of obstructive coronary disease amenable to revascularisation. A 2024 updated meta-analysis of randomised trials in chronic coronary syndromes has now demonstrated that percutaneous coronary intervention in addition to optimal medical therapy significantly reduces cardiovascular death (HR 0.81, 95% CI 0.71–0.93), reduces angina severity and improves quality of life compared with medical therapy alone [8].

In patients with incidental CAC, a substantial proportion will harbour obstructive stenoses, particularly when CAC is moderate-severe, and current guidelines already support anatomical or functional testing in intermediate-to-high risk asymptomatic individuals when CAC is known to be elevated. Future protocols may therefore reasonably propose that selected inpatients with newly identified incidental CAC, especially if ordinal scoring is implemented as “moderate” or “severe”, merit prompt outpatient CT coronary angiography—rather than isolated gated calcium/CAC scoring—to exclude flow-limiting disease that could derive mortality benefit from revascularisation. This approach would align with the evolving evidence base shifting the treatment paradigms toward anatomy-guided revascularisation in chronic syndromes, in conjunction with optimal medical therapy.

Shortcomings in risk factor management may stem from several factors. First, non-gated scans do not provide Agatston scores, leading to qualitative reporting that may be dismissed as non-actionable [11,40]. SCCT/STR guidelines advocate semi-quantitative reporting; none, mild, moderate and severe, on all non-gated chest CTs to facilitate risk stratification [11]. The BSCI/BSCCT/BSTI consensus provides guidance on reporting incidental CAC with a visual ordinal score and suggested report text: “Mild/Moderate/Severe coronary artery calcification, indicating the presence of coronary artery disease. If the patient has associated symptoms, recommend management as per chest pain guidelines (e.g., NICE CG95, SIGN 151). If the patient is asymptomatic, consider reviewing modifiable cardiovascular risk factors and managing as per guidelines for primary prevention (e.g., NICE CG 181)” [41]. It emphasises no upper age limit for reporting CAC and avoiding terms like “normal for age” [41]. Second, inpatient settings prioritise acute care over preventive measures [30].

Notifying patients of incidental CAC offers benefits, as in the NOTIFY-1 project [14]. CAC improves risk estimation beyond existing equations, and visualising atherosclerosis motivates adherence [14,24]. Notification can increase statin initiation, consistent with trials like EISNER and DANCAVAS [14,26,27]. The 2024 ESC Guidelines support integrating incidental CAC with the RF-CL model, a pre-test probability tool combining age, sex, symptoms, and risk factors, to estimate obstructive CAD likelihood, tripling very low-risk categorizations without losing accuracy [2]. Exercise ECG can reclassify low-likelihood patients, though CTCA is preferred for accuracy [2]. Combined with CAC in the CACS-CL model, it refines stratification and guides functional testing in intermediate cases [2]. Our opportunistic approach leverages existing imaging for cost-effective screening.

Limitations include the single-centre design, small sample size, and reliance on electronic medical documentation, which may underestimate measurements of patient assessments. CAC was reported qualitatively without severity grading, precluding outcome correlations. Future prospective studies could evaluate interventions like automated alerts or flowcharts [41]. This study is limited by its performance of only descriptive statistics; no inferential analyses were performed due to the exploratory nature of the study and absence of comparable risk-factor data in patients without reported CAC. Strengths include the real-world inpatient cohort, comprehensive data, detailed risk factor assessment, and identification of gaps for interventions.

In summary, incidental CAC on non-gated CT thorax represents an underutilised opportunity for CVD prevention in inpatients. Implementing standardised reporting and management protocols per SCCT/STR and ESC recommendations could bridge this gap and reduce long-term CVD burden [2,11]. A prospective randomised trial is now warranted to determine whether a strategy of routine CT coronary angiography following incidental CAC detection in asymptomatic or minimally symptomatic inpatients improves long-term cardiovascular outcomes, statin adherence, and cost-effectiveness compared with usual care. Such a study could randomise patients with moderate-severe incidental CAC to immediate CTCA-guided management versus guideline-directed medical therapy alone, with primary endpoints of major adverse cardiovascular events and cardiovascular mortality at 5 years.

## 5. Conclusions

One-fifth of medical inpatients in our study had a new finding of CAC on thoracic imaging, yet cardiovascular risk factors like LDL and HbA1c were assessed in less than half, with no functional cardiac testing performed. This highlights a valuable opportunity to optimise risk factors and evaluate for further testing in patients with incidental CAC on non-cardiac CTs. Raising awareness through education and tools like management flowcharts could facilitate better care.

## Figures and Tables

**Table 1:** Demographic information for N = 36 persons aged between 40 and 75 with an incidental finding of Coronary Artery Calcification on CT imaging.

Category	Subcategory	Value (n [%] or Mean [SD])
Demographics		
Total Patients	-	36
Age	-	63 (SD 8) years
Gender	Male	26 (72.2%)
	Female	10 (27.8%)
Observed Risk Factor Prevalence		
	Hypertension	23 (63.9%)
	Dyslipidemia	12 (33.3%)
	Current Smoker	14 (38.9%)
	Diabetes	4 (11.1%)
CT Indications		
	CTPA for suspected pulmonary embolism	20 (55.6%)
	CT thorax for suspected malignancy	10 (27.8%)
	CT aorta for suspected dissection	3 (8.3%)
	CT thorax for suspected sepsis	3 (8.3%)

**Table 2:** Cardiovascular risk factor assessment according to ESC guidelines, for N = 36 persons aged between 40 and 75 with an incidental finding of Coronary Artery Calcification on CT imaging.

Assessment Metric	Blood Pressure (n/N [%])	LDL Cholesterol (n/N [%])	Smoking Status (n/N [%])	HbA1c (n/N [%])
Recorded in Medical Records	36/36 (100.0%)	14/36 (38.9%)	36/36 (100.0%)	16/36 (44.4%)
Suboptimal Control ^a^	23/36 (63.9%)	12/14 (85.7%)	14/36 (38.9%)	4/16 (25.0%)
Treated if Suboptimal ^b^	6/23 (26.1%)	6/12 (50.0%)	5/14 (35.7%)	1/4 (25.0%)

^a^ Defined as office systolic blood pressure >140 mm Hg or diastolic blood pressure > 90 mm Hg on 2 occasions; LDL cholesterol > 1.8 mmol/L; active smoking; HbA1c > 48 mmol/mol or >6.5%; N = total patients or subgroup as applicable. LDL = low-density lipoprotein. ^b^ Treatment rates are expressed as proportion of patients with suboptimal values who received intervention. For smoking status, “suboptimal control” = current smoking; “treated” = documented smoking cessation advice/pharmacotherapy/referral.

## Data Availability

The data presented in this study are available on request from the corresponding author, H.C.T., upon reasonable request. The data are not publicly available due to privacy and ethical restrictions to protect patient confidentiality.

## Supplementary Materials

The following supporting information can be downloaded at: https://www.mdpi.com/article/10.3390/jcdd12120480/s1, Table S1: STROBE checklist.
