# Supplementary material for: Coronary Artery Calcification on Non-Cardiac Gated CT Thorax Scans: A Single Tertiary Centre Retrospective Observational Study
Source: J Cardiovasc Dev Dis. 2025 Dec 4;12(12):480. doi: 10.3390/jcdd12120480 (PMC12734173; doi:10.3390/jcdd12120480)
Supplement: Supplementary file 1 [file jcdd-12-00480-s001.zip › jcdd-3940024-supplementary.pdf]

STROBE Checklist:

Item No	Recommendation	Reported in Manuscript (Section/Page if applicable)
1	(a) Indicate the study's design with a commonly used term in the title or the abstract	Yes (Title: "Single Tertiary Centre Retrospective Observational Study")
	(b) Provide in the abstract an informative and balanced summary of what was done and what was found	Yes (Abstract: Includes Background, Methods, Results, Conclusions)
2	Explain the scientific background and rationale for the investigation being reported	Yes (Introduction)
3	State specific objectives, including any prespecified hypotheses	Yes (Introduction, last paragraph: Aims to explore prevalence and evaluate management)
4	Present key elements of study design early in the paper	Yes (Methods: "Single-centre retrospective observational study" and "observational cohort study")
5	Describe the setting, locations, and relevant dates, including periods of recruitment, exposure, follow-up, and data collection	Yes (Methods - Study Design and Population: Mater Misericordiae University Hospital, Dublin, Ireland; admissions February 1 to March 31, 2025; data collected May to June 2025)
6	(a) Cohort study—Give the eligibility criteria, and the sources and methods of selection of participants. Describe methods of follow-up	Yes (Methods - Study Design and Population: Age 40-75, unscheduled medical admissions via ED, non-cardiac gated CT thorax; exclusion for known IHD; retrospective review of electronic health records, no prospective follow-up described but implied through record review for downstream investigations)
	(b) Cohort study—For matched studies, give matching criteria and number of exposed and unexposed	Not applicable (No matching)
7	Clearly define all outcomes, exposures, predictors, potential confounders, and effect modifiers. Give diagnostic criteria, if applicable	Yes (Methods - Data Collection: Defines CAC presence, risk factors like hypertension (SBP >140 or DBP >90 on two occasions), LDL (>1.8 mmol/L), smoking (active), HbA1c (>48 mmol/mol or >6.5%); outcomes include risk factor assessment, treatment initiation, and downstream investigations like stress tests or angiography)
8	For each variable of interest, give sources of data and details of methods of assessment (measurement). Describe comparability of assessment methods if there is more than one group	Yes (Methods - Data Collection: From patient documents, letters, discharge summaries, CT reports, blood results; CT interpreted by consultant radiologists using standard protocols; qualitative CAC assessment; no multiple groups for comparability)

Item No	Recommendation	Reported in Manuscript (Section/Page if applicable)
9	Describe any efforts to address potential sources of bias	Yes (Methods - Data Collection: "Using a standardized protocol to ensure consistency"; Ethical Considerations: Data anonymized; Limitations in Discussion acknowledge reliance on documentation which may underestimate assessments)
10	Explain how the study size was arrived at	Yes (Methods - Study Design and Population: Consecutive general medical inpatients aged 40-75 undergoing CT thorax during February-March 2025; Results: Total 186 identified)
11	Explain how quantitative variables were handled in the analyses. If applicable, describe which groupings were chosen and why	Yes (Methods - Data Analysis: Continuous as means; categorical as frequencies and percentages; no groupings beyond categorical definitions)
12	(a) Describe all statistical methods, including those used to control for confounding	Yes (Methods - Data Analysis: Descriptive statistics only; no inferential or confounding control as exploratory)
	(b) Describe any methods used to examine subgroups and interactions	Not applicable (No subgroups or interactions examined)
	(c) Explain how missing data were addressed	Yes (Methods - Data Analysis: "Missing data for cardiovascular risk factors were addressed by conducting analyses solely on available records without imputation, as the study focused on descriptive summaries of documented assessments.")
	(d) Cohort study—If applicable, explain how loss to follow-up was addressed	Not applicable (Retrospective design; no prospective follow-up or loss described)
	(e) Describe any sensitivity analyses	Not applicable (None performed)
13	(a) Report numbers of individuals at each stage of study—eg numbers potentially eligible, examined for eligibility, confirmed eligible, included in the study, completing follow-up, and analysed	Yes (Results: 186 underwent scans; 53 with CAC reported; 17 excluded for known IHD; 36 analyzed)
	(b) Give reasons for non-participation at each stage	Yes (Results: Exclusions for known IHD)
	(c) Consider use of a flow diagram	No (A flow diagram illustrating the participant selection process was initially prepared but ultimately omitted from the manuscript, as the straightforward nature of the inclusion and exclusion criteria, along with the small number of stages involved, rendered it redundant and did not enhance clarity beyond the textual description provided.

Item No	Recommendation	Reported in Manuscript (Section/Page if applicable)
14	(a) Give characteristics of study participants (eg demographic, clinical, social) and information on exposures and potential confounders	Yes (Results: Table 1 for demographics and indications; Table 2 for risk factors)
	(b) Indicate number of participants with missing data for each variable of interest	Yes (Results: For risk factors, e.g., LDL checked in 39%, HbA1c in 44%, implying missing for others)
	(c) Cohort study—Summarise follow-up time (eg, average and total amount)	Not applicable (No prospective follow-up; retrospective record review)
15	Cohort study—Report numbers of outcome events or summary measures over time	Yes (Results: No exercise stress tests booked; 1 coronary angiogram booked; risk factor assessments and treatments in Table 2)
16	(a) Give unadjusted estimates and, if applicable, confounder-adjusted estimates and their precision (eg, 95% confidence interval). Make clear which confounders were adjusted for and why they were included	Yes (Results: Descriptive percentages only, no adjusted estimates or CIs as no inferential statistics)
	(b) Report category boundaries when continuous variables were categorized	Yes (Methods: Defined thresholds for risk factors, e.g., LDL >1.8 mmol/L)
	(c) If relevant, consider translating estimates of relative risk into absolute risk for a meaningful time period	Not applicable (No risk estimates)
17	Report other analyses done—eg analyses of subgroups and interactions, and sensitivity analyses	Not applicable (None reported)
18	Summarise key results with reference to study objectives	Yes (Discussion: Summarizes prevalence and management gaps)
19	Discuss limitations of the study, taking into account sources of potential bias or imprecision. Discuss both direction and magnitude of any potential bias	Yes (Discussion: Limitations section - single-centre, small sample, reliance on documentation may underestimate, qualitative CAC without grading)
20	Give a cautious overall interpretation of results considering objectives, limitations, multiplicity of analyses, results from similar studies, and other relevant evidence	Yes (Discussion and Conclusions: Interprets findings in context of guidelines and other studies, cautious on opportunities and needs)
21	Discuss the generalisability (external validity) of the study results	Yes (Discussion: Single-centre, real-world inpatient cohort; suggests broader implications but notes limitations)
22	Give the source of funding and the role of the funders for the present	Yes (Funding statement: "This research received no specific grant from any funding

Item No	Recommendation	Reported in Manuscript (Section/Page if applicable)
	study and, if applicable, for the original study on which the present article is based	agency in the public, commercial, or not-for-profit sectors.")
